# Epigenetics of Delirium and Aging: Potential Role of DNA Methylation Change on Cytokine Genes in Glia and Blood Along With Aging

**DOI:** 10.3389/fnagi.2018.00311

**Published:** 2018-10-23

**Authors:** Gen Shinozaki, Patricia R. Braun, Benjamin W. Q. Hing, Andrew Ratanatharathorn, Mason J. Klisares, Gabrielle N. Duncan, Sydney S. Jellison, Jonathan T. Heinzman, Yasunori Nagahama, Liesl Close, Sayeh Sabbagh, Brian J. Dlouhy, Matthew A. Howard, Hiroto Kawasaki, Hyunkeun R. Cho

**Affiliations:** ^1^Department of Psychiatry, Carver College of Medicine, University of Iowa, Iowa City, IA, United States; ^2^Department of Psychiatry and Behavioral Sciences, Johns Hopkins University, Baltimore, MD, United States; ^3^Department of Epidemiology, School of Public Health, Columbia University Medical Center, Columbia University, New York, NY, United States; ^4^Department of Neurosurgery, University of Iowa, Carver College of Medicine, Iowa City, IA, United States; ^5^Department of Biostatistics, College of Public Health, University of Iowa, Iowa City, IA, United States

**Keywords:** delirium, epigenetics, DNA methylation, cytokine, tNF-alpha, aging

## Abstract

**Background:** Delirium in elderly patients is common and dangerous. Major risk factors include aging and exogenous insults, such as infection or surgery. In animal models, aging enhances pro-inflammatory cytokine release from microglia in response to exogenous insults. The epigenetic mechanism DNA methylation (DNAm) regulates gene expression and changes with age. Older individuals may have methylation changes that influence the increased cytokine upon insult, but the degree to which aging affects DNAm of cytokine genes is not fully understood.

**Methods:** The relationship between DNAm and aging of pro-inflammatory cytokine genes (TNF-alpha, IL1-beta, IL-6) was investigated using methylation array data in two cohorts. Brain and blood samples were collected from a neurosurgery cohort (NSG) of 21 subjects who underwent brain resection. A second cohort, the Grady Trauma Project (GTP), included blood samples from 265 subjects.

**Results:** In the NSG cohort, a significant negative correlation between age and DNAm in brain was found at a CpG in IL-6. With the GTP dataset, significant negative correlations between age and DNAm were seen at most of the CpGs in TNF-alpha. Also, TNF-Alpha expression increases with age. These GTP DNAm correlations were also nominally significant in NSG blood samples. In neuronal negative NSG brain tissue, a similar negative trend was observed.

**Conclusions:** With aging, a decrease in DNAm of cytokines gene CpGs in glia and blood was seen. As this can affect their expression, additional research is needed to fully elucidate the role of DNAm in aging and how it may influence the pathogenesis of delirium.

## Introduction

Although delirium in hospitalized elderly patients is common and dangerous, it is underdiagnosed and undertreated (Inouye, 2006; Inouye et al., 2014). It is estimated that 11.8 million patients over the age of 65 who are hospitalized annually have a minimal 15–20% chance of developing delirium (2–3 million cases a year). Delirium can add over $60,000 in healthcare costs per patient, this leads to costs to the healthcare system of over $150 billion per year in the U.S. alone (Inouye et al., 2014). Extensive efforts have been made to develop screening tools that can be easily administered [e.g., the Confusion Assessment Method (CAM) (Inouye et al., 1990), CAM-ICU (Ely et al., 2001, 2004), and the Delirium Rating Scale-revised 98 (DRS-R-98; Trzepacz et al., 2001)]. Nevertheless, delirium remains seriously underdiagnosed and undertreated (Ely et al., 2004; Spronk et al., 2009). In busy hospitals, the current screening methods have been shown to have suboptimal sensitivity (38–47%) in intensive care units (Van Eijk et al., 2011; Nishimura et al., 2016). Given that our society is aging, it is becoming increasingly important to predict which patients are at risk for delirium. This will require the identification of biomarkers of delirium risk.

### Elevated cytokines among delirium patients: human data

Because infection or surgical insult commonly trigger delirium, it has been hypothesized that delirium pathophysiology is a consequence of inflammation and inflammatory cytokines. Dillon et al. showed an association between levels of an inflammatory marker, C-reactive protein (CRP), and delirium (Dillon et al., 2017), and they also reported that the duration and severity of delirium can be predicted by high CRP levels (Vasunilashorn et al., 2017). Other groups reported associations of delirium with high levels of Galectin-3 and CRP among women in postpartum intensive care units (Zhu et al., 2017). A pro-inflammatory cytokine, IL-6, was elevated after surgery among patients with delirium compared to controls (Liu et al., 2013; Vasunilashorn et al., 2015). Thus, accumulating evidence from human studies suggests that inflammation may play key roles in the pathogenesis of delirium, but an understanding of why delirium is associated with an enhanced inflammatory response is lacking.

### Enhanced cytokines among aged animals in response to exogenous insults: animal model

**Figure 1:**
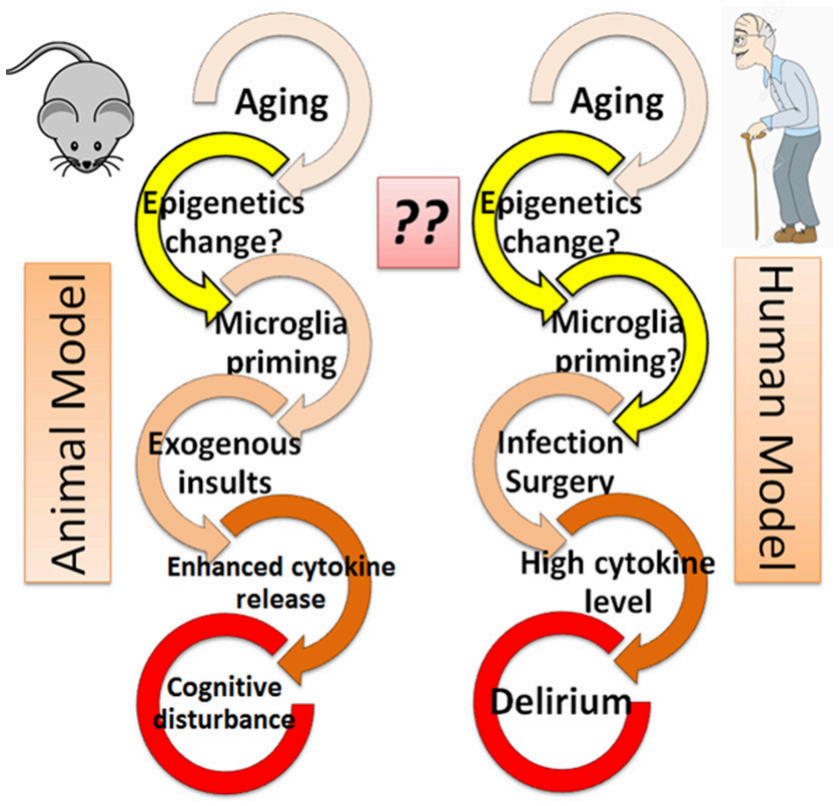
Epigenetics hypothesis–the missing link in the cascade from aging to delirium? Both animal and human models are shown. The role of epigenetics (yellow arrows) is not well understood and thus warrants further investigation.

A role for cytokines in cognitive disturbance is also supported by animal studies. Behavioral studies have suggested that aged rats experience cognitive disturbance after exposure to lipopolysaccharide [LPS; (Chen et al., 2008; Henry et al., 2009)] or surgical insult (Hovens et al., 2013; Wang et al., 2015). This is thought to be caused in part by an elevation of inflammatory cytokines, including IL-1beta, IL-6, and TNF-alpha, both in blood and in cerebrospinal fluid (Hovens et al., 2013; Wang et al., 2015), especially among aged animals. Such a mechanism is supported by the observation that the addition of agents that block these cytokine pathways prevents the effects of cognitive disturbances among aged animals after infection (Barrientos et al., 2012) or surgical insult (Frank et al., 2010). These animal models support roles for aging in conjunction with exogenous insults, such as infection (LPS) or surgical incision, in the pathogenesis of “delirium-like” cognitive disturbance that is mediated in party by inflammatory responses. Indeed, this combination is consistent with clinical characteristics of delirium, where the prevalence of delirium is higher in elderly patients in the hospital who have experienced infection or surgical intervention (Figure 1).

### Heightened microglial activation in response to exogenous insults among aged animals: similarity to delirium

In response to exogenous insults, microglia cells have been shown to release more cytokines in the brain of aged animals than in young animals, indicating activated microglia may contribute to the pathogenesis of cognitive decline in aged animals (Dilger and Johnson, 2008; Schreuder et al., 2017). Although the microglia response is not demonstrated in human studies, the similarity of cognitive decline in both animal models and human patients who experience exogenous insults (Figure 1) suggests that an enhanced microglial response to surgical or infectious insult may play a role in the pathophysiology of delirium (Dilger and Johnson, 2008; Schreuder et al., 2017).

### Epigenetics hypothesis: the missing link?

Although animal models support the notion that microglia are activated in response to exogenous insult (infection or surgery) in the context of aging, why aged animals have an enhanced cytokine release remains unclear. It is possible that the microglia response is molecularly preprogrammed prior to exposure to exogenous insult, and that this programming changes with aging. Aging is known to greatly influence gene expression in the brain (Kang et al., 2011), and such changes in gene expression are tightly regulated by epigenetic modifications, including DNA methylation (DNAm; Zampieri et al., 2015), histone modifications (Gong et al., 2015), and micro RNAs (Danka Mohammed et al., 2017). Indeed, DNAm pattens in brain have been shown to change dynamically throughout the human lifespan (Numata et al., 2012), and epigenetic processes involved in aging have been well studied (Day et al., 2013; Sen et al., 2016).

It is possible that aging-related epigenetic modifications in microglia enable an increased cytokine release in aged animals by altering the control of gene expression, and that this contributes to the pathophysiology of delirium. These epigenetic changes associated with aging could also occur in blood, where cytokines are released by monocytes and other blood cell types. Because delirium can be triggered by exogenous insults such as infection or surgery that cause inflammation in peripheral sites outside of the brain, epigenetic control of cytokine release from peripheral blood cells could also be partially responsible in the pathophysiology of delirium. Models with animals and cell cultures have shown that aging and LPS exposure affect cytokine production associated with DNAm changes (Green et al., 2015; Matt et al., 2016). Aged mice had decreased methylation in the promoter of *IL-1beta* in microglia at baseline and after LPS exposure in comparison to young mice. This decrease in DNAm was associated with heightened mRNA, IL-1β production, and prolonged sickness behavior (Matt et al., 2016). In another study, fibroblast taken from cows at different ages (5 or 16 months of age) were exposed to LPS, and the older cows showed an enhanced pro-inflammatory response with an increase in IL-6, IL-8, and TNF-alpha production, together with hypomethylation at promoter regions of those genes (Green et al., 2015).

To the best of our knowledge, however, no human study has investigated the role of epigenetics in delirium. Thus, it is crucial to investigate the role of epigenetics in delirium in human subjects. Our central hypothesis is that age-associated DNAm change in cytokine genes occurs in the general population, but in patients susceptible to delirium, DNAm is more significantly altered. Thus in response to exogenous insults in individuals who have differential methylation, cytokine release is more enhanced both peripherally (i.e., from monocytes in blood) and centrally (i.e., from microglia in brain), leading to delirium. Before investigating this broad hypothesis, it is necessary to determine if and in which specific CpGs/genes the aging process alters DNAm among cytokine genes in the general population.

In the present report, we used samples from two different cohorts to examine the association between aging and DNAm of cytokine genes. One cohort was a set of patients with medically intractable epilepsy from whom neurosurgically resected brain tissue and blood samples were collected simultaneously [neurosurgery cohort (NSG)]. Additionally, blood samples with DNAm and expression data were used from an independent, large cohort of 265 subjects from the Grady Trauma Project (GTP) (Smith et al., 2011).

## Methods and materials

### Study subjects and sample collection for NSG cohort

A more detailed overview of study participants and sample collection process has been described previously (Braun et al., 2017; Shinozaki et al., 2017). Briefly, 21 subjects with medically intractable epilepsy undergoing neurosurgery were recruited for this study between March 2014 and April 2017 at the University of Iowa Hospitals and Clinics. This study was approved by the University of Iowa's Human Subjects Research Institutional Review Board. Written informed consent was obtained, and whole blood samples were collected in EDTA tubes, saliva with the Oragene DISCOVER™ kit (DNA Genotek Inc., OGR-500), and buccal tissue with swabs (Puritan, 25-1506 1PF TT MC). Resected brain tissue samples were immediately stored and transported on dry ice, and a portion of each brain region was sent to pathology. All samples were stored at −80°C. Typically, blood samples were taken at the end of surgery in the operating room, and saliva and buccal swabs were collected within 2 days after the operation. FACS was performed as previously described (Braun et al., 2017; Shinozaki et al., 2017).

### Sample processing and epigenetics platform

Methylome assays were performed as previously described. Briefly, genomic DNA from saliva, buccal, and whole blood were isolated with the MasterPureTM DNA extraction kit (Epicenter, MCD85201). DNA was bisulfite-converted using the EZ DNA Methylation™ Kit (Zymo Research, D5002). The Infinium HumanMethylationEPIC BeadChip™ Kit (Illumina, WG-317-1002) was used to analyze DNAm of 21 subjects with brain, blood, saliva, and buccal samples. Raw data was processed using the R packages RnBeads and Minfi (Aryee et al., 2014; Fortin et al., 2017); this enables quality control checks, data filtering, and normalization of the data in addition to differential methylation analyses (Aryee et al., 2014; Assenov et al., 2014; Fortin et al., 2017).

### Statistical analysis

All statistical analyses were performed in R (R Core Team, 2013). DNAm correlation was calculated with Pearson's correlation using the average methylation for each tissue. The correlation of cytokine genes was performed with CpGs from those genes present on the array. The degree of correlation between aging and DNAm was calculated for each CpG in the cytokine genes tested. The correlation coefficient and its significance level was calculated with Spearman's test.

### Data from GTP cohort

Detailed sample collection for the Grady Trauma Project can be found in Smith et al. (2011). Illumina HumanMethylation450 DNAm data was processed according to a previously described pipeline (Ratanatharathorn et al., 2017). Briefly, the Infinium protocol was assessed at each step by visual inspection of control probes, after which background normalized beta values, methylated signals, unmethylated signals, and detection *p*-values were exported to R (Ihaka and Gentleman, 1996). Based on the R package CpGassoc, low-intensity samples (probe detection call rates < 90% and an average intensity value less than half the overall median or 2,000 arbitrary units) and proves with detection *p*-values > 0.001 were removed (Barfield et al., 2012). Sex chromosome cross-hybridizing probes were also removed (Chen et al., 2013). Type I and Type II probes were normalized with Beta Mixture Quantile Normalization after which the ComBat procedure was run twice to remove chip then position effects while controlling for gender and PTSD status (Leek et al., 2012; Teschendorff et al., 2013). Prior to analysis, β-values were logit transformed into M-values (Du et al., 2010).

For the gene expression analysis, RNA was extracted from whole blood collected in Tempus tubes at about 8:30 a.m., for all participants. All samples had Bioanalyzer RNA Integrity Number (RIN) 6. Probes were considered sufficiently expressed if they had a detection *p*-value of < 0.01 in 5% of the samples. In total 15,877 probes met these criteria. The array was log2 transformed and normalized using the Supervised Normalization Method (Mecham et al., 2010).

## Results

### Patient demographics

#### NSG study

We analyzed brain and peripheral tissue samples from 21 NSG subjects, and among them, seven were female and the average age was 31 years (SD ± 16.4). The resected and analyzed brain regions included the temporal cortex (11 individuals), the frontal cortex (four individuals), the hippocampus (four individuals), and the occipital area (two individuals). Fluorescence-activated cell sorting (FACS) was performed on brain tissues to separate cells positive for a neuronal marker, resulting in six neuronal-positive (neuron) samples and 13 neuronal-negative (glia) ones with sufficient DNA quantity for analysis.

#### GTP study

Detailed information about the GTP cohort has been described previously (Smith et al., 2011). Briefly, the total sample size with available DNAm and expression data is 265, 71% were female, and the average age was 42 years (SD ± 12) at the time of collection. Ninety-four percent of samples were received from African American individuals and 5% were received from Caucasian individuals.

### DNA methylation from NSG brain and blood samples

**Figure 2:**
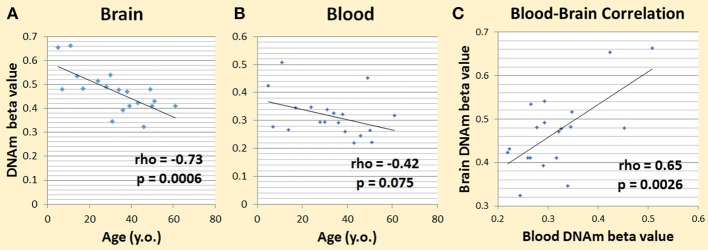
Preliminary data showing DNAm changes with age. **(A)** Changes in brain IL-6 cg23731304 DNAm and age correlated significantly. **(B)** Changes in blood IL-6 cg23731304 DNAm showed a similar trend as brain with aging. **(C)** Blood and brain DNA.m at IL-6 cg23731304 correlated significantly.

We first used our dataset from the NSG study (Braun et al., 2017; Shinozaki et al., 2017) to see if DNAm patterns among cytokine genes are correlated with aging in the brain. We looked at DNAm levels and their association with age across subjects (age rage 5–62 years) in brain tissue resected during neurosurgery. We chose the pro-inflammatory cytokine genes, *IL-1beta, IL-6*, and *TNF-alpha*, to test, which includes a total of 52 CpGs. Six CpGs correlated with aging at nominal significance (*p* < 0.05). Given that one in 20 tests would be significant by chance alone, in 52 CpGs we expected to have 2.6 that are significant by chance. Our top hit was in *IL-6* (cg23731304), which had a rho of −0.73 and a *p*-value of 0.00060 (Figure 2A). This was significant even after correction for multiple testing at the level of 0.05/52 = 0.00096. We then looked at this specific CpG and its DNAm trend in blood, and we found that its rho was −0.42 and the *p*-value was 0.075 (Figure 2B). The correlation of DNAm between brain and blood at this CpG was also significant (rho = 0.65; *p*-value = 0.0026) (Figure 2C).

### DNA methylation and expression from GTP cohort, a total of 265 subjects' blood samples

**Figure 3:**
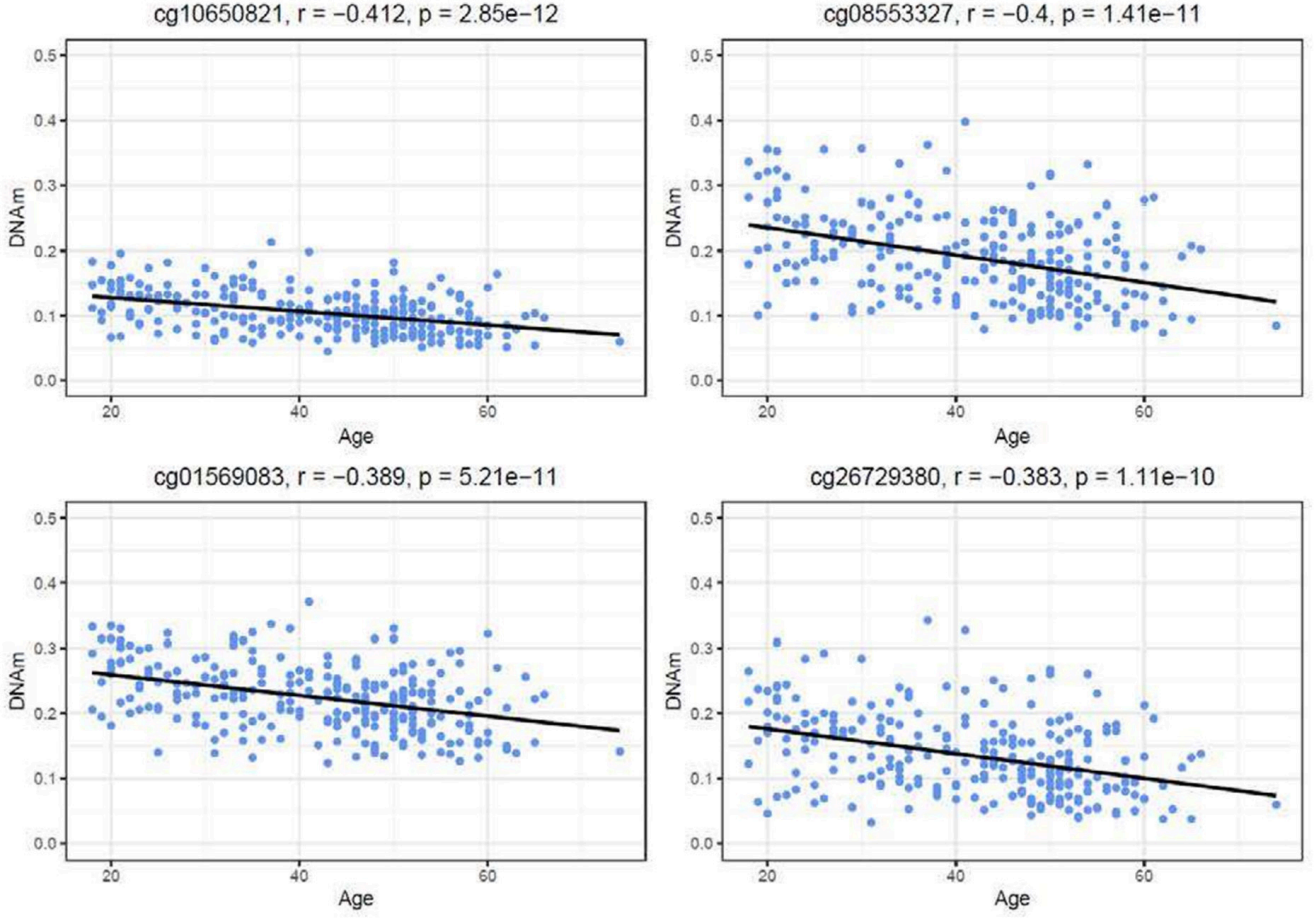
Correlations of age and DNAm levels from blood samples obtained from the GTP cohort. Top 4 hit CpG sites in TNFalpha and their DNAm level (beta value) in correlation with age (years) are shown.

**Figure 4:**
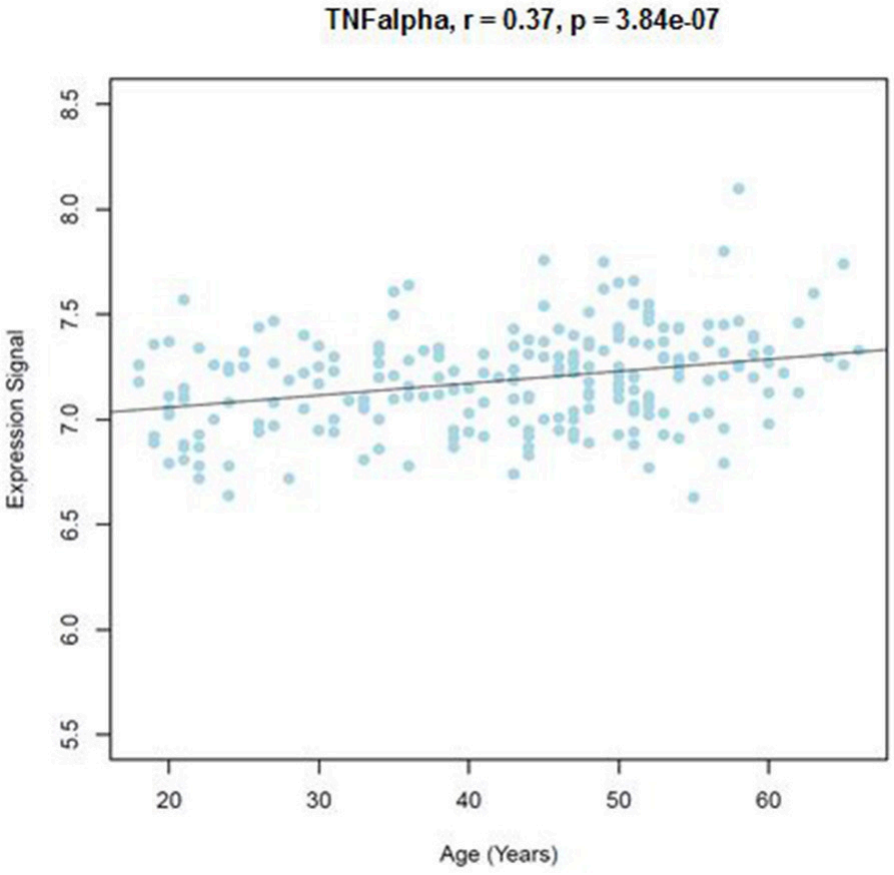
Correlations of age and TNFalpha expression level from blood samples obtained from the GTP cohort. TNFalpha expression level in correlation with age (years) is shown.

Similar to the NSG preliminary analysis, we sought to investigate the association between DNAm of *IL-1beta, IL-6, IL-8*, and *TNF-alpha* and aging among the GTP cohort. The dataset included 74 CpGs, and among them DNAm levels at 14 CpGs was associated with aging at nominal significance (*p* < 0.05). There were 38 CpGs that were significantly associated at nominal levels with aging, predominantly with *TNF-alpha* represented. In fact, all 27 CpGs in *TNF-alpha* were at least nominally significant (**Table 2**). Eight CpGs were significant at genome-wide significant levels (*p* < 5 × E-8), all in *TNF-alpha* (**Table 2**). The top hit was at cg10650821 (*r* = −0.41; *p*-value of 2.85 × E-12) (Table 1, and Figure 3). Moreover, when expression levels of these cytokines were tested for a subgroup of 215 subjects with available expression data, TNF-alpha expression was significantly positively associated with aging as expected (*r* = 0.37, *p* = 3.84 × E-7) (Figure 4).

**Table 1:** Correlations of age and DNAm levels from blood samples obtained from the independent GTP cohort.

**CPG info**				**GTP**		
**CpG**	**Chr**	**Position**	**Gene**	**N**	**r**	***p*-value**
cg10650821	6	31543686	TNF	265	−0.41	2.8544E-12
cg08553327	6	31543647	TNF	265	−0.40	1.4059E-11
cg01569083	6	31543289	TNF	265	−0.39	5.208E-11
cg26729380	6	31543655	TNF	265	−0.38	1.106E-10
cg04472685	6	31545473	TNF	265	−0.36	2.3131E-09
cg04425624	6	31543565	TNF	265	−0.35	4.8768E-09
cg21222743	6	31543545	TNF	265	−0.34	1.2343E-08
cg03037030	6	31543300	TNF	265	−0.33	2.7845E-08
cg21467614	6	31543638	TNF	265	−0.33	5.3752E-08
cg21370522	6	31543219	TNF	265	−0.32	7.6408E-08
cg10717214	6	31543557	TNF	265	−0.32	7.7839E-08
cg12681001	6	31543540	TNF	265	−0.32	8.6829E-08
cg11484872	6	31543169	TNF	265	−0.31	2.3968E-07
cg09637172	6	31545252	TNF	265	−0.29	1.7632E-06
cg02137984	6	31545898	TNF	265	−0.29	1.8168E-06
cg15703690	7	22766992	IL6	265	0.27	1.0416E-05
cg26736341	6	31545342	TNF	265	−0.25	3.3831E-05
cg06825478	6	31546067	TNF	265	−0.25	4.1973E-05
cg15989608	6	31545321	TNF	265	−0.25	5.3427E-05
cg05952498	6	31545257	TNF	265	−0.25	5.4834E-05
cg20477259	6	31544960	TNF	265	−0.23	0.00016172
cg01360627	6	31544931	TNF	265	−0.23	0.00022076
cg23384708	6	31544934	TNF	265	−0.22	0.00036899
cg17755321	6	31546085	TNF	265	−0.21	0.00058042
cg17741993	6	31544694	TNF	265	−0.19	0.00145946
cg15106030	4	74528435	IL8	265	−0.19	0.00184398
cg19124225	6	31545836	TNF	265	−0.17	0.005378
cg20157753	2	1.14E+08	IL1B	265	0.17	0.00588717
cg07998387	7	22767571	IL6	265	−0.17	0.00603186
cg24452282	6	31542740	TNF	265	−0.17	0.0064941
cg23149881	2	1.14E+08	IL1B	265	−0.14	0.01833347
cg18007641	4	74641828	IL8	265	−0.14	0.02275773
cg04392234	4	74608458	IL8	265	−0.14	0.02666244
cg17067544	7	22765321	IL6	265	−0.14	0.02757188
cg06850158	4	74567419	IL8	265	−0.13	0.02829831
cg18553677	7	22701635	IL6	265	−0.13	0.03362868
cg27531490	6	31542459	TNF	265	−0.13	0.03493842
cg03310594	7	22704316	IL6	265	−0.12	0.04311163
cg11814672	4	74600153	IL8	265	−0.11	0.07068166
cg03246791	2	1.14E+08	IL1B	265	−0.11	0.0751379

*TNFalpha, IL-1beta, IL-6, and IL-8 were tested with a total of 74 CpGs. Orange-highlighted CpGs were also nominally significant at NSG blood samples. Blue-highlighted CpGs were also nominally significant (light blue) at NSG brain Neu (–) samples (glia) or showed trends (dark blue) in the same direction (see Table 2). Most of these significant CpGs are from TNFalpha, other than few exceptions (gray shading)*.

### NSG samples revisited, DNA methylation from blood, and facs-sorted brain

We sought to determine if the GTP top findings were also represented in the NSG study. Three of the top four hits (cg10650821, cg08553327, and cg26729380, all from *TNF-alpha*, highlighted in orange in Table 2) were correlated with aging at nominal significance in blood (*N* = 21), resulting in an independent cohort replicating the finding.

**Table 2:** Comparison of age and DNAm at 24 CpGs in TNF-alpha gene using glial, neuron, whole brain, and blood from six individuals.

**Comparison of various tissue types from 6 subjects with neuronal positive brain available**															
**Glial**				**Neuron**				**Whole brain**				**Blood**			
**Gene**	**cgid**	**Rho**	***p***	**Gene**	**cgid**	**Rho**	***p***	**Gene**	**cgid**	**Rho**	***p***	**gene**	**cgid**	**Rho**	***p***
tnf	cg04472685	−1	0.017	tnf	cg04472685	−0.7	0.233	tnf	cg04425624	−0.9	0.083	tnf	cg26729380	−1	0.017
tnf	cg26736341	−1	0.017	tnf	cg02137984	−0.6	0.350	tnf	cg08639424	−0.9	0.083	tnf	cg04425624	−0.9	0.083
tnf	cg02137984	−0.9	0.083	tnf	cg06825478	−0.6	0.350	tnf	cg10717214	−0.9	0.083	tnf	cg08553327	−0.9	0.083
tnf	cg15989608	−0.9	0.083	tnf	cg10717214	−0.3	0.683	tnf	cg20477259	−0.9	0.083	tnf	cg10650821	−0.9	0.083
tnf	cg20477259	−0.9	0.083	tnf	cg21370522	−0.3	0.683	tnf	cg08553327	−0.8	0.133	tnf	cg10717214	−0.9	0.083
tnf	cg23384708	−0.9	0.083	tnf	cg23384708	−0.1	0.950	tnf	cg26736341	−0.7	0.233	tnf	cg12681001	−0.9	0.083
tnf	cg06825478	−0.7	0.233	tnf	cg01569083	0	1.000	tnf	cg19648923	−0.6	0.350	tnf	cg19124225	−0.9	0.083
tnf	cg10717214	−0.7	0.233	tnf	cg03037030	0.1	0.950	tnf	cg21222743	−0.6	0.350	tnf	cg19648923	−0.9	0.083
tnf	cg19648923	−0.7	0.233	tnf	cg19648923	0.1	0.950	tnf	cg04472685	−0.5	0.450	tnf	cg21467614	−0.9	0.083
tnf	cg01360627	−0.6	0.350	tnf	cg21222743	0.1	0.950	tnf	cg10650821	−0.5	0.450	tnf	cg06825478	−0.7	0.233
tnf	cg12681001	−0.6	0.350	tnf	cg26729380	0.1	0.950	tnf	cg15989608	−0.5	0.450	tnf	cg21222743	−0.7	0.233
tnf	cg19124225	−0.6	0.350	tnf	cg19978379	0.2	0.783	tnf	cg21370522	−0.5	0.450	tnf	cg01569083	−0.6	0.350
tnf	cg01569083	−0.4	0.517	tnf	cg08553327	0.3	0.683	tnf	cg01569083	−0.4	0.517	tnf	cg02137984	−0.1	0.950
tnf	cg03037030	−0.4	0.517	tnf	cg08639424	0.3	0.683	tnf	cg26729380	−0.4	0.517	tnf	cg03037030	−0.1	0.950
tnf	cg04425624	−0.4	0.517	tnf	cg12681001	0.4	0.517	tnf	cg01360627	−0.3	0.683	tnf	cg21370522	−0.1	0.950
tnf	cg10650821	−0.4	0.517	tnf	cg15989608	0.6	0.350	tnf	cg03037030	−0.3	0.683	tnf	cg01360627	0	1.000
tnf	cg19978379	−0.4	0.517	tnf	cg19124225	0.6	0.350	tnf	cg06825478	−0.3	0.683	tnf	cg04472685	0	1.000
tnf	cg21222743	−0.4	0.517	tnf	cg04425624	0.7	0.233	tnf	cg12681001	−0.3	0.683	tnf	cg08639424	0	1.000
tnf	cg21467614	−0.4	0.517	tnf	cg10650821	0.7	0.233	tnf	cg21467614	−0.3	0.683	tnf	cg15989608	0	1.000
tnf	cg24452282	−0.4	0.517	tnf	cg21467614	0.8	0.133	tnf	cg24452282	−0.3	0.683	tnf	cg19978379	0	1.000
tnf	cg26729380	−0.4	0.517	tnf	cg01360627	0.9	0.083	tnf	cg23384708	−0.2	0.783	tnf	cg20477259	0	1.000
tnf	cg08553327	−0.3	0.683	tnf	cg24452282	0.9	0.083	tnf	cg02137984	−0.1	0.950	tnf	cg23384708	0	1.000
tnf	cg08639424	−0.3	0.683	tnf	cg20477259	1	0.017	tnf	cg19978379	−0.1	0.950	tnf	cg24452282	0	1.000
tnf	cg21370522	−0.3	0.683	tnf	cg26736341	1	0.017	tnf	cg19124225	0	1.000	tnf	cg26736341	0	1.000

*Orange highlights a negative correlation and blue highlights a positive correlation*.

Next, we tested if these associations of DNAm in *TNF-alpha* with aging in the GTP cohort were similarly present in our NSG brain tissues. We used data from just the six subjects who have samples from each tissue type (whole brain, neuronal-positive, neuronal-negative, and blood) to better compare the trend in correlation between age and DNAm at all 24 CpGs tested in the *TNF-alpha* gene. In both whole brain tissue and FACS-sorted neuronal-positive (neuron) cells, no trends were identified, but in FACS-sorted neuronal-negative (glial) cells nominal significant associations with aging were seen at cg04472685 and cg26736341 (*p* = 0.017, light blue highlight in Tables 1, 2), and similar trends were seen in next four top CpG sites (dark blue highlight in Tables 1, 2).

All 24 CpGs showed a negative correlation in glia (rho = −1~–0.3), whole brain (rho = −0.9~0), and blood (rho = −1~0), whereas neuronal-positive cells showed variable rho value ranges from negative to positive (rho = −0.7~1) (Table 2). This shows a contrast between glia and neuron cell types in trends of DNAm associating with aging, as well as the similarity in trends between glia and blood. Of note, though, the sample size was very small, which could limit the ability to detect statistically significant trends.

## Discussion

The NSG data from 21 samples showed that in human brain the DNAm level of one CpG at a promotor region of the pro-inflammatory cytokine gene (*IL-6*) decreases with age, which could potentially associate with an increase in *IL-6* expression. Also, the DNAm level of this CpG in *IL-6* is correlated between brain and blood, and in blood, the DNAm shows a similar trend with aging. These findings suggest that blood DNAm of this *IL-6* CpG has the utility of an epigenetic biomarker of age-related changes in brain. In fact, there is also a report that shows a low DNAm at a single CpG in *IL-6* is associated with heightened expression from blood obtained from human subjects (Nile et al., 2008).

The data from an independent cohort in the GTP cohort revealed an age-associated DNAm decrease along with aging at multiple CpGs from blood samples. The pattern is very consistent with the NSG cohort. The top signal showed a genome-wide significant *p*-value of 2.85 × E−12. Adjusting for multiple comparison correction of the 74 CpG sites tested, 28 CpGs were significant at 0.05/74 = 0.00068, as listed in Table 2 (24 CpGs from GTP). The majority of these findings from blood DNAm were in *TNF-alpha* and showed a decrease with aging, which is consistent with literature (Gowers et al., 2011). Moreover, expression of TNF-alpha was positively correlated with aging as expected based on negative correlation of DNAm with age. On the contrary, expression of other pro-inflammatory cytokines except *TNF-alpha* was not correlated with aging from this cohort. This could indicate that other cytokines are stabilized in certain range when human body is in stable condition. However, because of underline DNAm change along with aging, when individuals are exposed to exogenous insults, they are more prone to enhanced expression, which, after a certain threshold, could put them more at risk for delirium. In fact, relationship between direct measurement of TNF-alpha level and delirium has been controversial. In patients with delirium, multiple study reported that TNF-alpha remain not significant (De Rooij et al., 2007; Çinar et al., 2014; Brum et al., 2015). Of note, sample sizes of delirium cases in these reports are less than 100 (64 cases, 15 cases, and 17 cases, respectively), thus the result might have been underpowered. Also, controlling for the level of exogenous insults can be difficult confounding factors to control. These conflicting data suggests that cytokine level itself may not work as a reliable biomarker of the risk of delirium, and DNAm may potentially provide a better underline risk factor for delirium.

The *TNF-alpha* results from the GTP were further interrogated in the NSG cohort, and it was shown that DNAm in almost all CpGs in *TNF-alpha* decrease with advanced age in blood. Furthermore, DNAm in *TNF-alpha* CpGs in FACS-sorted neuronal-negative tissue showed a similar decrease with aging. One CpG (cg15989608) was found to be nominally significantly associated with aging even with a limited sample size of 13 cases. This CpG was also significantly associated with aging from blood samples among the GTP cohort with a *p*-value 5.34 × E−5. When the NSG data was compared among six subjects who have samples from whole brain, glia, neuron and blood, a negative correlation was seen for most of *TNF-alpha's* 24 CpGs in glial, whole brain, and blood tissues, whereas neuronal-positive cells showed a wider range of correlations. This similar trend in glia and blood in *TNF-alpha* supports our hypothesis that glial cells (including microglia) and blood cells (including monocytes) could have similar DNAm changes in association with aging among pro-inflammatory cytokine genes.

It is possible that the trends we saw in DNAm decreasing with aging were due to the fact that DNAm in general tends to decrease with aging; however, as different tissues varied in their aging trends, this indicates the findings were not generalized. Thus, it is possible that age-associated decreases in DNAm level among pro-inflammatory cytokine genes are more dominant among glia and blood cells, as compared to neuronal cells.

Strengths of this report include its unique use of both blood and brain samples, in addition to FACS-sorted brain tissue, to investigate epigenetic changes in cytokine genes associated with aging. This approach using FACS-sorted brain could then compare neuron and glia cell types to find their distinct patterns of DNAm change with aging. Another strength is the use of two independent cohorts for the analysis, which allowed us to identify a persistent decrease of DNAm in *TNF-alpha* in two distinct cohorts. In fact, the data indicates that the result presented here can be potentially useful not only for delirium research, but for investigation of other age-related disorders, especially those where inflammatory process is playing a role.

Limitations of this report include the brain DNAm investigation is limited to 21 samples from the NSG project, with an even smaller sample size of FACS-sorted brain tissue. However, our correlation results between brain and blood at specific cytokine genes suggest that DNAm from blood could be potentially a good surrogate for that of brain, especially from glia. Unfortunately, we also did not have specific blood cell type DNAm data, such as monocytes, or specific glial cell types, such as microglia, which warrants further investigation because of our hypothesis about the role of microglia. As there is a lack of research in the epigenetics of delirium to guide the investigation of specific gene targets, we tested pro-inflammatory cytokine genes based on their potential role in delirium. In addition to these genes, however, a genome-wide approach would be of use for future studies that investigate epigenetic mechanisms specific to delirium instead of aging more broadly.

The data presented here provide evidence of DNAm-associated changes in cytokine genes with aging in blood and brain tissues, especially among glial cells. As it is possible that individuals susceptible to delirium have exacerbated or dysregulated changes in DNAm in these genes, this research provides a basis for further testing our hypothesis of epigenetic change in delirium. For this goal, we have initiated a study to collect samples from subjects with and without delirium to test genome-wide DNAm difference.

An identification of epigenetic biomarkers associated with delirium could potentially improve current practice of medicine and surgery. For example, where possible, patients who are identified to be at high risk for delirium may postpone surgery until the risk diminishes, or preventative measure could be employed to have patients closely monitored after surgery to minimize dangerous outcomes. This would allow for limited resources in the hospital to be allocated more efficiently.

In summary, we propose a hypothesis for the role DNAm on cytokine genes in delirium pathophysiology. Specifically, we hypothesize that with aging, there is a decrease in DNAm in pro-inflammatory cytokine genes, which could make them more prone to be expressed, especially in response to exogenous insults, such as infection or surgery. Thus, such inflammatory response with heightened cytokine levels could potentially lead to delirium. This preliminary investigation of DNAm associated with aging in pro-inflammatory cytokines showed that DNAm in *TNF-alpha* and *IL-6* CpGs are negatively correlated with aging both in brain and blood tissues. For *TNF-alpha*, glia and blood showed similar DNAm trends with aging in contrast to neuron tissues. Importantly, this study shows that DNAm levels in cytokine genes are associated with aging, but it is necessary to determine if aged individuals susceptible to delirium have different methylation in contrast to aged individuals who do not develop delirium after exogenous insults.

## Ethics statement

This study was carried out in accordance with the recommendations of the University of Iowa IRB with written informed consent from all subjects. All subjects gave written informed consent in accordance with the Declaration of Helsinki. The protocol was approved by the University of Iowa IRB.

## Author contributions

GS conceptualized the study and designed the study. PB, BH, JH, MK, GD and SJ processed samples. PB, BH, AR and HC analyzed the data. YN, LC, BD, MH, and HK obtained the samples. SS organized the samples and data. GS drafted the initial manuscript. PB, BH, AR and HC revised the manuscript. All authors reviewed the manuscript and approved the content.

### Conflict of interest statement

GS has disclosed that he is a founder of Predelix Medical LLC. The remaining authors declare that the research was conducted in the absence of any commercial or financial relationships that could be construed as a potential conflict of interest. The reviewer AK and handling Editor declared their shared affiliation at the time of the review.
